# Uptake and Acceptability of Oral HIV Self-Testing among Community Pharmacy Clients in Kenya: A Feasibility Study

**DOI:** 10.1371/journal.pone.0170868

**Published:** 2017-01-26

**Authors:** Peter M. Mugo, Murugi Micheni, Jimmy Shangala, Mohamed H. Hussein, Susan M. Graham, Tobias F. Rinke de Wit, Eduard J. Sanders

**Affiliations:** 1 Kemri-Wellcome Trust Research Programme, Kilifi, Kenya; 2 Ministry of Health, Mombasa, Kenya; 3 University of Washington, Seattle, Washington, United States of America; 4 University of Amsterdam, Amsterdam, the Netherlands; 5 University of Oxford, Headington, United Kingdom; University of Pittsburgh, UNITED STATES

## Abstract

**Background:**

While HIV testing and counselling is a key entry point for treatment as prevention, over half of HIV-infected adults in Kenya are unaware they are infected. Offering HIV self-testing (HST) at community pharmacies may enhance detection of undiagnosed infections. We assessed the feasibility of pharmacy-based HST in Coastal Kenya.

**Methods:**

Staff at five pharmacies, supported by on-site research assistants, recruited adult clients (≥18 years) seeking services indicative of HIV risk. Participants were offered oral HST kits (OraQuick®) at US$1 per test. Within one week of buying a test, participants were contacted for post-test data collection and counselling. The primary outcome was test uptake, defined as the proportion of invited clients who bought tests. Views of participating pharmacy staff were solicited in feedback sessions during and after the study.

**Results:**

Between November 2015 and April 2016, 463 clients were invited to participate; 174 (38%) were enrolled; and 161 (35% [95% Confidence Interval (CI) 31–39%]) bought a test. Uptake was higher among clients seeking HIV testing compared to those seeking other services (84% vs. 11%, adjusted risk ratio 6.9 [95% CI 4.9–9.8]). Only 4% of non-testers (11/302) stated inability to pay as the reason they did not take up the test. All but one tester reported the process was easy (29%) or very easy (70%). Demand for HST kits persisted after the study and participating service providers expressed interest in continuing to offer the service.

**Conclusions:**

Pharmacy HST is feasible in Kenya and may be in high demand. The uptake pattern observed suggests that a client-initiated approach is more feasible compared to pharmacy-initiated testing. Price is unlikely to be a barrier if set at about US$1 per test. Further implementation research is required to assess uptake, yield, and linkage to care on a larger scale.

## Introduction

HIV testing and counselling (HTC) is a key entry point for treatment as prevention [1]. It is also an HIV prevention intervention in its own right, as it may result in more consistent condom use [2] and adoption of other risk reduction strategies [3]. Recognition of the central role of HTC in HIV prevention has intensified its implementation in recent years [4]. In Kenya, the proportion of adults 15–64 years who have ever tested increased from 37% in 2007 to 70% in 2012 [5], and to 80% in 2014 [6]. However, despite this increase in first-time testing, 53% of HIV-infected adults in a 2012 national survey were unaware they were infected [7], hence were not on treatment and may have continued high risk behaviour. This highlights the need for innovative HTC approaches.

A quarter to a half of patients in developing countries seek care at community pharmacies [8–10]. Reasons why patients may prefer pharmacies over health facilities include: perceived non-serious nature of illness, convenient placement of premises, lower cost of treatment, quicker services, greater perceived privacy, and responsiveness of pharmacy personnel to client demands [8, 11–13]. With regard to stigmatized illnesses such as sexually transmitted infections (STIs), pharmacies may provide a quick “no-questions-asked” service [14]. Given this care-seeking pattern, community pharmacies constitute a unique channel for reaching target populations with disease control interventions.

Interest to expand the involvement of community pharmacies in HIV prevention is growing [15] [16], particularly with regard to HIV testing [17–19]. Pharmacy-based HIV testing has been implemented successfully in the United States [20–22], but has not been assessed in sub-Saharan Africa. We recently demonstrated the feasibility of referring pharmacy clients for HIV testing in Kenya [23]. A quarter of targeted clients in that study took up testing at a referral site [23]. We hypothesized that uptake would be higher if the service was available on site, for example, through provision of self-test kits.

Kenyan national HTC guidelines recommend HIV self-testing (HST) as part of a comprehensive HIV testing programme [24, 25]. Licensing of the first self-test kit is imminent and roll-out plans are underway [25, 26]. Formative studies indicate that Kenyans mostly prefer to obtain HST kits through public health facilities, but also expect to be able to obtain them from pharmacies [27]. A number of studies are assessing HST distribution through health facilities, including antenatal care clinics [28, 29] and pre-exposure prophylaxis sites [30].

We aimed to determine HST uptake among at-risk pharmacy clients in Coastal Kenya, assess acceptability and willingness to pay, and document experiences and views of participating pharmacy staff.

## Materials and Methods

### Study setting

The study was conducted in a rapidly urbanizing area located 16 km north-east of Mombasa (total population ∼100 000), with very active nightlife and beach tourism spots [31]. Pharmacies in the study area are typically small to medium-sized businesses, employing two people and serving 60 clients per day on average [31].

### Stakeholder engagement

In the months leading up to the study, the concept was discussed with key stakeholders in HIV prevention and pharmacy practice, including the National AIDS and STI Control Programme (NASCOP), the Pharmacy and Poisons Board (PPB), the Pharmaceutical Society of Kenya (PSK), and community pharmacy practitioners (n = 4). All consulted stakeholders expressed support for the project and gave suggestions for improving the study and possible scale-up.

### Pharmacy selection

Based on experience gained in a previous study [23], we targeted pharmacies that were registered with the PPB, had higher client flow compared to other pharmacies in the area, had adequate private space (private room or private corner in the dispensary), and whose management gave approval. Of 17 pharmacies assessed, we engaged five; four were not interested, four did not receive management approval, two did not have adequate private space, and two had low client flow.

### Study population

We targeted adult pharmacy clients (≥18 years) seeking services indicative of HIV risk, including: HIV testing, STI treatment, malaria/ fever treatment, condoms, lubricants, sexual performance enhancers, combined oral contraceptives, emergency contraceptives, HIV post-exposure prophylaxis (PEP), pregnancy testing, needles and syringes. Eligibility criteria included: active mobile phone, ability to read English or Kiswahili, and not HIV infected (self-report).

### Test kit used

We used the OraQuick® rapid HIV 1/2 antibody test (OraSure Technologies, Bethlehem, PA, USA), a lateral-flow, immuno-chromatographic, second-generation, oral-fluid assay detecting antibodies to HIV-1 and HIV-2. It has a sensitivity of 89.7% and specificity of 98.0% when used by lay persons in Kenya [32]. The study kits were purposed for over-the-counter distribution and un-assisted self-testing. Each kit came with all the necessary components packaged in a pocket-size plastic pouch.

Tests were procured through a local agent at US$9.80 per test, supplied to pharmacies free of charge, and sold to participants at a subsidized price of KSh 100 (~US$1) per test–the median maximum price that participants in a recent questionnaire study conducted in Mombasa and Siaya counties of Kenya were willing to pay [33].

### Procedures

#### Staff training and responsibilities

We trained pharmacy service provider (PSPs) and research assistants (RAs) through initial off-site meetings (~2 hours), weekly on-site visits with data-driven feedback, and 2-monthly off-site refresher trainings. PSPs were pharmacists (degree holders), pharmaceutical technologists (diploma holders), and pharmacy assistants (certificate holders and apprentices), while RAs (n = 2) were experienced HTC counsellors engaged specifically for the study. PSPs were responsible for participant recruitment. RAs were responsible for consenting, counselling and data collection. Each RA was responsible for two pharmacies at a time; pharmacies supported by the same RA were less than three minutes’ walk apart. During the last three months, following training and practical experience, PSPs also participated in consenting, counselling (pre-test) and data collection (pre-test).

#### Participant recruitment

Eligible clients were given a brief introduction and invited to participate by the attending PSP. Clients who were in a hurry were issued a numbered card containing brief study information and asked to return as soon as possible. Enrolled clients were encouraged to invite their partners. Following feedback from PSPs, promotional wall posters were displayed inside study pharmacies during the last 1.5 months of the study.

#### Pre-test data collection

For all clients invited at a pharmacy, basic data were recorded in a pre-printed booklet, including: gender, age, service sought, date and time of visit, outcome of invitation, and initials of PSP. After consenting, date of birth and phone number were recorded. The phone number was dialled to check that it was recorded correctly and that it was active. Reasons for not enrolling or not buying a test were documented. A pre-test questionnaire was administered, asking about previous testing and views about HST (see supplementary S1 Text).

#### Pre-test counselling

Counselling and test instructions were closely aligned to the national guidelines [24] and the test kit package insert (see supplementary S2 Text). Counselling included: basic HIV education, benefits of testing and disclosure, and options for further support. Participants were advised to seek confirmatory testing if self-test result was positive or invalid; and repeat testing in three months if exposed to HIV recently (in previous three months). They were informed that the test was meant for home testing and that they were not required to report the test results. Participants who bought a test were provided a test-aid card containing the RA’s telephone number and a list of health facilities and HTC centres that had agreed to offer confirmatory testing.

#### Post-test data collection and follow-up

A post-test questionnaire was administered within a week of purchasing the test either on phone for those who tested at home or face-to-face for those who chose to test at the pharmacy and those who returned to the pharmacy after testing. The interview included questions on testing experience and views about the test approach (see supplementary S3 Text). For those who voluntarily reported positive results, additional counselling was provided and confirmatory testing and linkage to care documented.

#### Feedback sessions

We held two feedback sessions: two months to the end of the study and two weeks after the end of the study. All participating PSPs and RAs were invited. The sessions were moderated by the first and second authors. Topics included: thoughts on quantitative study results, experiences during the study, and things to consider during a scale-up. Discussions were audio-recorded, transcribed verbatim, and compiled into a narrative summary.

### Statistical considerations and data analysis

The primary outcome was HST uptake: the proportion of invited clients who enrolled and purchased a test. We predicted a 45% uptake based on our previous study on test referrals [23]. To be able to measure this uptake with a 5% margin of error at the 95% confidence level, we needed a sample of at least 381 clients. We aimed to invite 400 clients and enrol 180 clients (45%).

Data cleaning, recoding and analysis was conducted using Stata® Version 13 (StataCorp, College Station, Texas, USA). Proportions were calculated for client characteristics, test uptake, and acceptability. For Likert-type variables collected pre- and post-test, change was assessed using the Wilcoxon matched-pairs signed-ranks test. Associations between client characteristics and HST uptake were assessed using log binomial regression [34]; variables collected on enrolled clients only were not included.

### Ethical considerations

The study was approved by the Kenya Medical Research Institute (KEMRI) Scientific and Ethics Review Unit (KEMRI/SERU/CGMR-C/0011/3089). Participants provided written informed consent. Participants received KSh 50 phone airtime after completing the post-test questionnaire. Service providers received KSh 50 for each client enrolled by the RA and KSh 100 if they enrolled the client themselves. Those who attended off-site meetings received KSh 500 transport reimbursement. Proceeds from sale of tests, at KSh 100 per test, were granted to pharmacy owners.

## Results

### Flow of invited and enrolled clients

**Fig 1:**
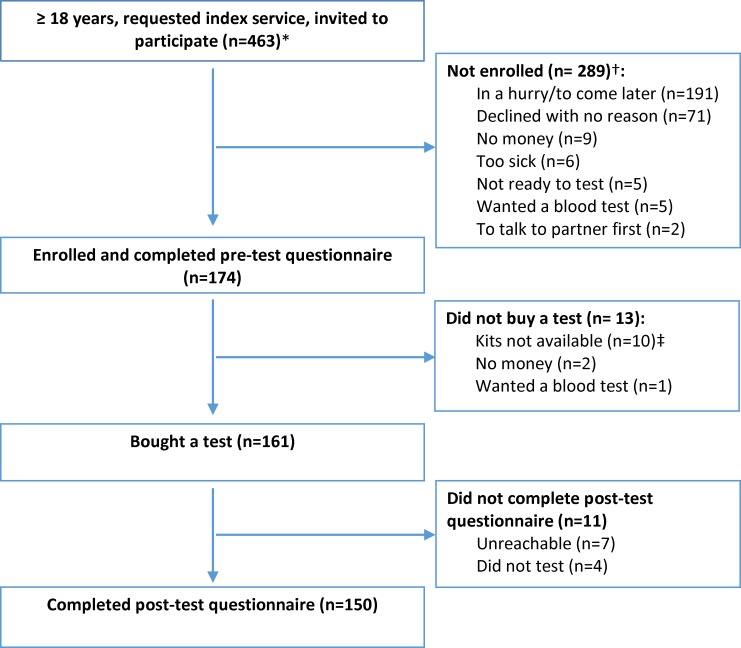
Flow of clients. *Three participants returned to the study sites and retested; data from their repeat visits are not included. †Includes 245 clients who declined immediately and 44 who declined after a more in-depth briefing. ‡There was a delay in delivery of test kits from the supplier; ten enrolled clients did not return after tests became available despite several reminders.

From Nov 2015 to Apr 2016, 463 clients were invited to participate and 174 (38%) were enrolled (Fig 1).

### Characteristics of invited and enrolled clients

Table 1 presents basic characteristics of invited and enrolled clients. There were statistically significant differences between invited and enrolled clients with regard to the service sought (p<0.001) and whether service was sought for self or someone else (p = 0.006), but not with regard to age and gender (p>0.05).

**Table 1:** Basic characteristics of invited and enrolled clients.

Characteristic		Invited clients	Enrolled clients
		N (%)	N (%)
**Number**		463 (100%)	174 (100%)
**Age (years):**			
	18–24	117 (25%)	46 (26%)
	25–35	220 (48%)	83 (48%)
	>35	126 (27%)	45 (26%)
**Gender:**			
	Male	225 (49%)	85 (49%)
	Female	238 (51%)	89 (51%)
Literacy*:			
	Both English and Kiswahili	—	160 (92%)
	Kiswahili only	—	14 (8%)
**Service sought:**			
	HIV testing†	149 (32%)	130 (75%)
	Malaria treatment	69 (15%)	9 (5%)
	Emergency contraceptives	67 (14%)	10 (5%)
	Pregnancy testing	62 (13%)	10 (6%)
	Sexual performance enhancers	40 (9%)	2 (1%)
	Condoms	29 (6%)	4 (2%)
	Combined oral contraceptives	22 (5%)	5 (3%)
	STI treatment	19 (4%)	2 (1%)
	Other‡	6 (1%) ‡	2 (1%)
**For whom the service was sought:**			
	Self	423 (91%)	167 (96%)
	Other	40 (9%) §	7 (4%)

* Assessed for enrolled clients only.

† 54 clients (12% of invited and 31% of enrolled) were specifically seeking HST after hearing about the study from other participants (n = 25) or other people (n = 29).

‡ Lubricants (n = 4), PEP (n = 1) needles (n = 1).

§ Partner (n = 23), family member (n = 13), friends (n = 2), for resale (n = 2).

Of 174 enrolled clients, 36 (21%) had self-tested for HIV before (Table 2), using tests obtained from pharmacies (n = 25), health facilities (n = 6), HTC centers (n = 3), and friends (n = 2). Most of the clients reported using blood-based rapid tests meant for professional use (n = 28); a few could not remember the type of test used (n = 8). At pharmacies, tests were bought at about KSh 50–150 (anecdotal reports). Sixty (34%) had self-tested for other health conditions, including pregnancy (n = 43), malaria (n = 12), and diabetes (n = 5).

**Table 2:** Previous self-testing experience and HIV test history among enrolled clients, N = 174.

Characteristic		N (%)
**Ever self-tested for HIV before**		36 (21%)
Ever heard about oral HIV self-testing*		12 (7%)
**Ever self-tested for other health conditions**		60 (34%)
**Ever tested for HIV with sex partner**		94 (56%)
**Number of times ever tested for HIV:**		
	0	5 (3%)
	1	23 (13%)
	2	26 (15%)
	3	30 (17%)
	4 or more	90 (52%)
**Duration since the last test:**		
	Never tested	5 (3%)
	More than 12 months	50 (29%)
	Less than 12 months	119 (68%)
**Main reason for last test:**		
	To understand illness/ symptoms	47 (28%)
	Advice from a doctor/ health provider	31 (18%)
	Possible exposure to HIV	25 (15%)
	To plan the future/ take charge of own health/ getting married	21 (12%)
	Encouraged by sex partner	19 (11%)
	Encouraged by friend	14 (8%)
	Employment/ travel/ donating blood	9 (5%)
	Curiosity	3 (2%)
**Where last test was done:**		
	Private health facility†	64 (38%)
	Government health facility	57 (34%)
	Mobile testing service	21 (12%)
	HTC centre	20 (12%)
	Self-testing	7 (4%)
**Main reason for where last test was done:**		
	Easily accessible	107 (64%)
	Did not choose–was seeking other services	28 (17%)
	Confidentiality/ privacy	19 (11%)
	Presence of skilled staff and/ or quality services	14 (8%)

* From other study participants (n = 9) or media and internet (n = 3).

† Incudes for-profit, non-governmental organization (NGO), community-based organization (CBO), and faith-based facilities.

### HST uptake and test outcomes

Overall HST uptake was 35% [95% CI 31–39%]. From the multivariable analysis, uptake was associated with product sought and study period after promotional wall posters; but not with client age, client gender, pharmacy visited, time of pharmacy visit, or gender of service provider (see supplementary S1 Table). Uptake among clients seeking HIV testing was 84% (125/149) compared to 11% (36/314) among clients seeking other services (adjusted risk ratio 6.9 [95% CI 4.9–9.8]).

Only 4% (11/302) stated inability to pay as the reason they did not take up the test. Of 149 clients seeking HIV testing who did not take up HST (n = 24, 16%), reasons were: in a hurry (n = 14), wanted a blood test (n = 6), kits not available (n = 3), no money (n = 1).

Among 174 enrolled clients, the likelihood of buying a test was higher among those who could read both English and Kiswahili compared to those who could read only Kiswahili (94% Vs 79%, p = 0.04, bivariable analysis); no other significant factors were found in this sub-sample.

For 161 clients who bought a test, 66% took away the test for home testing, while 34% tested at the pharmacy. One study pharmacy had a separate consultation/ lab room which was used for pre-test counselling and in-pharmacy testing. For the other four, this was done in the dispensary. However, owing to a high number of participants at one of the four pharmacies, a tent was erected at the back during the last 3 months of the study to accommodate pre-test counselling and in-pharmacy testing.

Pre-test counselling was provided mostly by RAs (79%), but also by PSPs (21%). According to post-study estimates, pre-test counselling took about five minutes for clients who had tested before and ten minutes for those who had never tested; in-pharmacy testing took twenty minutes, inclusive of counselling.

Seven percent of clients who bought a test (11/161) did not complete the post-test questionnaire: seven were unreachable and four did not test. Reasons for not testing were: travelled and forgot kit at home, changed mind, kit trashed by partner who insisted they test at a health facility, and kit “taken” by a friend.

All 150 testers revealed their test results to the RA, and 94 (63%) reported disclosing the results to someone else, including partners (n = 57), family members (n = 19), and friends (n = 17). Two clients tested positive, for a screening prevalence of 1.3% [95% CI 0.2–4.8%]; both were in-pharmacy testers and both reported enrolling in care.

All but one tester reported the process was easy (29%) or very easy (70%). The one tester reporting the process was difficult received invalid results (no test line) and was advised to re-test at a testing centre of her choice.

### HST acceptability among enrolled clients

At the pre-test interview, a majority of participants agreed (35%) or strongly agreed (59%) that HST kits should be made available to the general public. Explanations given by ten (6%) respondents who disagreed (n = 2) or were undecided (n = 8), but who nevertheless bought tests, included: “not sure how others will handle the results” (n = 3), “I haven’t heard about it before (n = 2), “it’s a personal thing” (n = 2), and “lack of a counsellor” (n = 1). Explanation given by one participant who disagreed and did not buy a test was that “people may not be able to handle positive results”. Agreement level increased during post-test interviews, with 23% agreeing and 74% strongly agreeing (p = 0.002).

A similarly large majority agreed (49%) or strongly agreed (47%) that pharmacies would be the best place to access HST. Three respondents who disagreed were concerned that not everyone would be able to pay, and the other three did not explain. Agreement level increased during post-test interviews, with 32% agreeing and 65% strongly agreeing (p = 0.002).

The main advantage of HST mentioned was privacy followed by personal empowerment. Risk of distress or harm when testing in the absence of a counsellor was the main disadvantage cited (Table 3).

**Table 3:** Main advantages and disadvantages of HIV self-testing cited at the pre-test interview, N = 174.

**What do you think is the main advantage of self-testing for HIV? *(select one)***	**N (%)**
Privacy/ anonymity/ confidentiality	91 (52%)
Personal empowerment / taking charge of one’s own health	44 (25%)
No pricking/ painless	14 (8%)
Saves cost/ no fare to the HTC centre or clinic	12 (7%)
Saves time / no waiting in queues	9 (5%)
I don’t see any advantage	4 (2%)
**What do you think would be the main disadvantage of self-testing for HIV?**	**N (%)**
Absence of counsellor when testing/ increased distress after a positive result / increased possibility of self-harm or suicide/ increased possibility of harming others	74 (43%)
I don’t see any disadvantage	25 (14%)
Illiterate people may not be able to use the method	25 (14%)
Difficulties/ mistakes in performing the test or interpreting the results	20 (12%)
Reduced chance of disclosure/ enrolment in care	13 (8%)
Testing others without their consent	11 (6%)
Production of fake or poor quality test kits	3 (2%)
Some people might not afford the kits	1 (1%)
The kits may be affected by the environment and be inaccurate	1 (1%)

Except for one tester who was unhappy with the arrangement of pictures in the package insert, all testers stated they would like to conduct HST again in future, and that they were likely (19%) or very likely (80%) to recommend self-testing to a friend, partner or family member.

When asked for additional information, most suggestions and concerns cited were related to test process and access (Table 4).

**Table 4:** Additional suggestions and concerns regarding pharmacy HIV self-testing.

Category	Detail
Test process (n = 13)	Ensure counsellors are available in pharmacies (n = 6)
	Reduce the 20-minute time for reading results (n = 2)
	Make the test device smaller so that children can use without difficulties (n = 2)
	Reduce number of questions asked before purchase of kit (n = 1)
	Improve the arrangement of pictures in the instructions leaflet (n = 1)
	Make the kit more accurate to eliminate need for confirmatory testing (n = 1)
Access (n = 10)	Avail the kits in normal retail shops to increase access (n = 3)
	Advertise the service widely (n = 2)
	Avail the kits in hospitals so that they can be free (n = 2)
	Make the kits free in pharmacies (n = 1)
	Avail the kits to pregnant mothers so that they can easily and privately test themselves (n = 1)
	The kit should be distributed door-to-door (n = 1)
Other (n = 4)	Provide ARVs in pharmacies (n = 2)
	Avail a similarly easy-to-use test for diabetics (n = 1)
	Concerned about the kit being misused by those who want to infect others since it can give false negative results if the tester is on ARVs (n = 1)

### HST provider views and experiences

Of 22 PSPs serving in the five pharmacies during the study period (2 pharmacists, 16 pharmaceutical technologists, and 4 pharmacy assistants), 18 (9 male [M] and 9 female [F]) participated in offering HST, and eight (6M, 2F) participated in the feedback sessions. The first session included seven PSPs (5M, 2F), while the second one included six PSPs (4M, 2F), all pharmaceutical technologists. Below is a narrative summary of the discussions.

#### Demand for pharmacy HST existed before the study and persisted after the end of the study

PSPs concurred with quantitative data suggesting high prevalence of pharmacy HST in the study area: 14% of participants (25/174) reported previous self-testing using tests bought in pharmacies (see above). When we noted that pharmacy-based HIV testing was non-existent during our 2011 survey [31], client demand was cited as the main reason for the change in practice:

*“Clients come asking…like they ask today*, *tomorrow…so you’ll be tempted to keep the kits in your pharmacy”* (Male PSP)

Demand for HST kits persisted after the end of the study and PSPs expressed strong interest to continue offering the service:

*“Up to now people are coming*, *disturbing*, *they want the test*! *…They say ‘You’ve just started*, *and you have gone away*, *what’s this now*?*’ …You can bring the services we handle ourselves*.*”* (Male PSP)

*“…even some clinical officers*, *they are demanding for it [the self-test kit]*, *that at least if they get some few they can start practising it in their clinics*.*”* (Male PSP)

#### Clients seeking services other than HIV testing were more challenging to engage

While clients seeking HIV testing were easily convinced to buy the Oraquick® test, other clients were more challenging. PSPs described difficult encounters with clients, which sometimes made them selective in their engagement:

*“At that time*, *they don’t want to be told about HIV”* (Male PSP)

*“One asked me ‘do I look like I have HIV*?*’ …I just smiled and let them go*.*”* (Female PSP)

*“You have to determine who you talk to because an arrogant client doesn’t give you time*. *Someone will walk in looking moody*, *if you try talk to them*, *they may even leave you talking to yourself*. *They may even not buy what had brought them there…so you lose a client*.*”* (Female PSP)

In particular, male PSPs found male clients most challenging, especially those seeking sexual performance enhancers, while female PSPs were challenged by female clients, especially those seeking emergency contraceptives:

*“[those seeking performance enhancers]…they just come in a hurry “Give me*!*” and vanish”* (Male PSP)

*“I didn’t get many challenges with the enhancers*, *but with the e-pills [emergency contraceptives]*. *I think it is the gender thing*. *For a man buying an enhancer from a man*, *it’s bringing down their ego… He thinks he is already judging him*. *And when a lady is buying an e-pill*, *they think I am judging them*. *…I ask the [male] RA to handle them”* (Female PSP)

It was felt that the general hurried nature of pharmacy clients negatively impacted uptake and pre-test counselling:

*“…Because even them they are in a hurry*. *If someone came for the Vega [performance enhancer] and you just try and give them like a lecture of 15 or 20 minutes… that won’t happen*.*”* (Male PSP)

Clients of perceived higher socioeconomic status were more challenging to engage:

*“When you try to approach them [well-off clients]*, *they mention big hospitals and say they have already tested*. *They tend to move away from the services that are being given to the community*. *They feel like they are somewhere else…socially*.*”* (Male RA, Pharmacy 2)

#### Privacy and availability of counsellors were the main reasons some clients chose to test at the pharmacy, not at home

*“Some were just doing it like a secret*, *they want to know themselves first before they can introduce the partner”* (Male PSP)

*“I think they expect that they will get instant counselling after the test*. *Unlike doing at home where you’re alone”* (Male PSP)

#### PSPs supported scale-up of pharmacy HST and suggested things to consider during a scale-up

There was strong support for wider implementation of pharmacy HST, but limited to registered pharmacies:

*“Yes*, *of course*! *…This will help the larger population…this will help people to know their status so at least to minimize the HIV transmission”* (Male PSP)

*“[I recommend] only registered pharmacies*. *Because you might give it to another pharmacy which doesn’t have a registered personnel or professional and the way he or she might handle the client*, *based on the results*, *it won’t be a good thing”* (Male PSP)

Advertisement and awareness creation to the general public was cited as a potential strategy to enhance uptake and “normalize” the service:

*“If we do a good advertisement regarding Oraquick® and it is readily available*, *that will attract people*. *You shouldn’t target only those coming for condoms and so on”* (Female PSP)

Some PSPs were enthusiastic about providing pre-test counselling, while others felt that it would be an added burden:

*“One way to improve uptake would be to equip PSPs to be able to administer the test*. *There are those guys who might feel more comfortable dealing with you because you have been with them for a long time*.*”* (Male PSP)

*“Me I‘ll need a counsellor… Sometimes we are busy*.*”* (Male PSP)

There was general agreement on the need for training, but concerns were cited regarding the feasibility of off-site training:

*“If you provide this kit*, *you should also maybe come up with a package in training the staff or the team on the ground…because a few maybe have the skills in counselling a client while others don’t*.*”* (Male PSP)

*“It depends on our bosses*, *others will accept [we go for training]*, *others will not*.*”* (Male PSP)

To address staff turnover, PSPs suggested a provider support system in addition to the training:

*“In case I’m no longer in [current pharmacy]…and the staff joining has no knowledge on how these kits work…can we have a special team*, *basically a team of counsellors who one can refer to or call immediately…for guidance*?*”* (Male PSP)

## Discussion

This study demonstrates the feasibility of pharmacy HST in a developing country setting. Uptake was high among pharmacy clients seeking HIV testing (84%), but very low among those seeking other services (11%), suggesting that a client-initiated approach would be more feasible compared to pharmacy-initiated testing. Only 4% of non-testers stated inability to pay as the reason they did not take up the test, suggesting that cost might not be a barrier if subsidized and set at about one dollar per test, as was the case in the study. Acceptability was high among testers and service providers. Privacy was predictably the most popular aspect of HST, but personal empowerment was also cited as an important benefit. A large majority of testers expressed interest in using the method again in future and a high likelihood of recommending it to others.

The low uptake among clients seeking services other than HIV testing is of concern. It is important to note that enrolment in the study was required before buying the test, hence the observed uptake may be confounded by willingness to participate in research. The low uptake may also be related to the fact that pharmacy-based HIV testing is a relatively new concept. Indeed, uptake increased towards the end of the study and PSPs suggested that greater public awareness could enhance uptake and normalize the service. However, similar to our earlier studies [23, 31], the low uptake, particularly among those seeking STI treatment or with symptoms compatible with acute HIV infection (e.g. clients requesting malaria treatment), highlights the need for training of pharmacy staff to ensure that at-risk clients are provided with HIV education and testing, as well as linkage to comprehensive HIV prevention and treatment services.

Findings from this study suggest that pharmacy HST may be in high demand in Kenya and that it is already being provided unofficially: one in five participants reported previous self-testing using blood-based rapid tests meant for professional use and demand for HST kits persisted after the end of the study. Official roll-out of pharmacy HST will ensure quality through provision of tests purposed for unassisted self-testing and through training and monitoring.

Price for the unofficial tests was reported to be US$1 on average, similar to the price of oral tests in the study, suggesting this could be a viable price for a scale-up. However, tests would need to be supplied to pharmacies free of charge or greatly subsidized. To prevent price hikes of subsidised tests, measures would need to be put in place, for example, printing the price on the package and broadcasting it in the media.

Similar to previous HST studies [35], testing for HIV in the absence of a counsellor was a prominent concern among participants in our study. About half of enrolled clients mentioned it as the main disadvantage and PSPs suggested that the need for post-test counselling may have been a major reason why one in three clients chose to test at the pharmacy and not at home. However, maintaining a dedicated counsellor in each pharmacy and post-test follow-up by pharmacies may not be feasible in a scale-up. Pharmacy HST providers should be equipped to provide pre-test counselling and self-testers should be provided with post-test counselling support such as printed information handed out with the test and a telephone hotline. Further research is needed to evaluate client and provider support mechanisms and to confirm the feasibility of pharmacy HST in the absence of a dedicated counsellor.

Although we did not exclude any client based on literacy, about one in ten participants could only read in Kiswahili. Innovative approaches will be needed to cater for illiterate clients. In-pharmacy testing, which could be an option, may not always be feasible or acceptable. At a minimum, test instructions will need to include illustrations, as was the case in the study. For HST kits distributed in Coastal Kenya, and perhaps the wider East African region, package inserts will need to be in both English and Kiswahili, as was the case in the study.

Our study had some limitations. First, since invited clients who declined participation did not complete a questionnaire, we were unable to assess their potential HST acceptability outside of a research study. Second, study pharmacies were selected purposively, hence findings may not be generalizable to all pharmacies in Kenya. This could be particularly true with regard to availability of private space for pre-test counselling and in-pharmacy testing. Greater regulatory enforcement plus adoption of professional self-regulation initiatives, such as the Green Cross and Pharmnet initiatives [36] [37], could address this concern. Third, the study design did not allow for assessment of self-testing performance in terms of test accuracy since participants who tested negative were not required to undergo confirmatory testing.

In conclusion, we found that pharmacy HST is feasible in Kenya and may be in high demand. The uptake pattern observed suggests that a client-initiated approach is more feasible compared to pharmacy-initiated testing. Price is unlikely to be a barrier if set at a nominal, subsidized price of US$1 per test. Further implementation research is required to assess uptake, yield, and linkage to care on a larger scale.

## Supporting Information

S1 Dataset Dataset. (CSV)

S1 Table Factors associated with HST uptake, based on multivariable analysis. (DOCX)

S1 Text Pre-test questionnaire. (PDF)

S2 Text Test kit package insert. (PDF)

S3 Text Post-test questionnaire. (PDF)
