# Supplementary material for: Uptake and Acceptability of Oral HIV Self-Testing among Community Pharmacy Clients in Kenya: A Feasibility Study
Source: PLoS One. 2017 Jan 26;12(1):e0170868. doi: 10.1371/journal.pone.0170868 (PMC5268447; doi:10.1371/journal.pone.0170868)
Supplement: S1 Table — (DOCX) [file pone.0170868.s002.docx]

**Table 3: Factors associated with HIV self-test uptake**

| **Factor** | | **N**  **Invited** | | **N**  **Who bought a test**  **(% of invited)** | **Bivariable analysis** | | **Multivariable analysis**  **(Full model)** | |
| --- | --- | --- | --- | --- | --- | --- | --- | --- |
|  |  |  |  |  | **Risk ratio**  **[95% Confidence interval]** | **P value** | **Adjusted risk ratio**  **[95% Confidence interval]** | **P value** |
| **Client age (years):** | | | | | | | | |
|  | 18-24 | | 117 | 43 (37%) | 1.1 [0.8-1.5] | 0.6 | -- | -- |
|  | 25-35 | | 220 | 75 (34%) | Ref | Ref | -- | -- |
|  | >35 | | 126 | 43 (34%) | 1.0 [0.7-1.4] | 1.0 | -- | -- |
| **Client gender:** | | | | | | | | |
|  | Male | | 225 | 78 (35%) | Ref | Ref | -- | -- |
|  | Female | | 238 | 83 (35%) | 1.0 [0.8-1.3] | 1.0 | -- | -- |
| **Product/ service sought:** | | | | | | | | |
|  | HIV testing | | 149 | 125 (84%) | 24.3 [3.6-167.1] | 0.001 | 23.1 [3.4-158.0] | 0.001 |
|  | Malaria treatment | | 69 | 8 (12%) | 3.4 [0.4-25.7] | 0.2 | 3.3 [0.4-25.0] | 0.3 |
|  | Emergency contraceptives | | 67 | 10 (15%) | 4.3 [0.6-32.3] | 0.2 | 4.3 [0.6-31.8] | 0.2 |
|  | Pregnancy testing | | 62 | 7 (11%) | 3.2 [0.4-25.0] | 0.3 | 3.4 [0.4-26.2] | 0.3 |
|  | Sexual performance enhancers | | 40 | 2 (5%) | 1.5 [0.1-15.2] | 0.8 | 1.8 [0.2-19.0] | 0.7 |
|  | Condoms | | 29 | 1 (3%) | Ref | Ref | Ref | Ref |
|  | Regular contraceptives | | 22 | 4 (18%) | 5.3 [0.6-44.0] | 0.1 | 5.2 [0.6-42.6] | 0.1 |
|  | STI treatment | | 19 | 2 (10%) | 3.1 [0.3-31.6] | 0.4 | 3.3 [0.3-33.7] | 0.3 |
|  | Other | | 6 | 2 (33%) | 10.0 [1.1-90.0] | 0.03 | 10.4 [1.2-90.8] | 0.03 |
| **For whom the product/ service was sought:** | | | | | | | | |
|  | Self | | 423 | 155 (37%) | 2.4 [0.7-31.4] | 0.02 | 1.0 [0.5-2.1] | 1.0 |
|  | Other | | 40 | 6 (15%) | Ref | Ref | Ref | Ref |
| **Pharmacy visited:** | | | | | | | | |
|  | 1 | | 186 | 68 (37%) | 1.6 [0.9-3.0] | 0.1 | 1.5 [1.0-2.6] | 0.1 |
|  | 2 | | 41 | 9 (22%) | Ref | Ref | Ref | ref |
|  | 3 | | 39 | 14 (36%) | 1.6 [0.8-3.3] | 0.2 | 1.4 [0.8-2.4] | 0.3 |
|  | 4 | | 98 | 47 (48%) | 2.2 [1.2-4.0] | 0.01 | 1.4 [0.8-2.4] | 0.4 |
|  | 5 | | 99 | 23 (23%) | 1.1 [0.5-2.1] | 0.9 | 1.3 [0.7-2.2] | 0.5 |
| **Time of pharmacy visit:** | | | | | | | | |
|  | Morning | | 116 | 37 (32%) | 1.2 [0.7-1.9] | 0.5 | -- | -- |
|  | Afternoon | | 178 | 67 (38%) | 1.4 [0.9-2.2] | 0.1 | -- | -- |
|  | Evening | | 102 | 39 (38%) | 1.4 [0.9-2.3] | 0.1 | -- | -- |
|  | Night | | 67 | 18 (27%) | Ref | Ref | -- | -- |
| **Gender of service provider:** | | | | | | | | |
|  | Male | | 198 | 90 (45%) | 1.7 [1.3-2.2] | <0.001 | 1.2 [1.0-1.5] | 0.08 |
|  | Female | | 265 | 71 (27%) | Ref | Ref | Ref | Ref |
| **Promotional wall posters:** | | | | | | | | |
|  | Before posters | | 366 | 97 (27%) | Ref | Ref | Ref | Ref |
|  | After posters | | 97 | 64 (66%) | 2.5 [2.0-3.1] | <0.001 | 1.2 [1.0-1.4] | 0.04 |
