# Supplementary material for: Uptake and Acceptability of Oral HIV Self-Testing among Community Pharmacy Clients in Kenya: A Feasibility Study
Source: PLoS One. 2017 Jan 26;12(1):e0170868. doi: 10.1371/journal.pone.0170868 (PMC5268447; doi:10.1371/journal.pone.0170868)
Supplement: S1 Text — (PDF) [file pone.0170868.s003.pdf]

PHARMACY SELF-TESTING STUDY

Pharmacy ID: _________ Client ID: ___________

Date *(dd/mmm/yyyy)*: ____ / _____ / ______

PRE-TEST QUESTIONNAIRE [DCF3]

Now I will ask you a number of questions about previous tests you may have taken and your views towards HIV self-testing provided through pharmacies. I request that you answer all the questions truthfully. If you do not want to answer any of the questions you may say so and I will move on to the next question

1. **Have you ever tested/ been tested for HIV?**
   1 - Yes *(Go to Q3)*
   2 - No

2. *[If not tested before]* **What is the main reason for not testing?** *(select one)* *(After this, Go to Q9)*
   1 - I don't think I have HIV/ I have no reason to test
   2 - I am afraid of learning a positive HIV status
   3 - It takes too much time/ I don't have time
   4 - I don't like needles/ testing procedure
   5 - Other, specify:________________

3. **How many times have you ever tested/ been tested for HIV?** 1 2 3 $\geq$4 times

4. **(a) Month and year when you last tested for HIV** *(mmm/yyyy)*: ______ / ______
   (b) Results: 0 – Not willing to disclose 1- NEG 2- POS 3 – indeterminate/ don't know

5. **(a) Where did you do the last test?**
   1 - Government hospital/ clinic
   2 - Private hospital/ clinic (including NGO, CBO and faith-based facilities)
   3 - VCT/ HTC center
   4 - Mobile testing service
   5 - Other, specify:____

   (b) What was the main reason for choosing this facility? *(select one)*
   0 - I didn't choose myself, I had gone for other services
   1 - Easily accessible
   2 - Confidential/ private/ discreet
   3 - Presence of skilled staff/ quality services/ information/ support
   4 - Other, specify:____

6. **What was the main reason/ motivation for testing?** *(select one)*
   0 - Employment/ travel requirement
   1 - Advice from doctor or health provider/ ANC invitation
   2 - Encouraged by friend or family member
   3 - Encouraged by sex partner
   4 - Possible exposure to HIV
   5 - To understand illness or symptoms that I had
   6 - To plan the future/ getting married/ take charge over one's health
   7 - Other, specify:____

7. **Have you ever tested with your sex partner?**
   1—Yes 2—No 3—Don't have a partner

8. **(a) Have you ever tested yourself for HIV in the past?** Yes No *(Go to Q9)*
   (b) Where did you get a test kit? 1 – Pharmacy 2 – other, specify: ____________________
   (c) What type of test kit was it? ____

9. **Before today, have you heard about the oral HIV self-test?** Yes No
   If yes, briefly describe: ____

10. **Have you ever tested yourself for other health conditions?** Yes No
    If yes, briefly describe: ____

11. **(a) How much do you agree or disagree with the statement:**

    "HIV self-test kits should be made available to the general public"?

1	2	3	4	5
Strongly disagree	*Disagree*	*Undecided*	*Agree*	*Strongly agree*

    Briefly explain: ____

12. **(a) Who specifically do you think need access to HST kits?** ____________________________

    (b) Briefly explain: ____

13. **What do you think is the <u>main advantage</u> of self-testing for HIV?** *(select one)*
    0 - I don't see any advantage
    1 - Privacy/ anonymity/ confidentiality
    2 - Personal empowerment / taking charge of one's own health
    3 - Saves cost/ No fare to the vct or clinic
    4 - No pricking/ painless
    5 - Saves time / no waiting in queues
    6 - Other, specify:____________________________

14. **What do you think would be the <u>main disadvantage</u> of self-testing for HIV?** *(select one)*
    0 - I don't see any disadvantage
    1 - Illiterate people may not be able to use the method
    2 - Difficulties/mistakes in performing the test or interpreting the results
    3 - Absence of counsellor when testing/ increased distress after a positive result / increased possibility of self-harm or suicide/ increased possibility of harming others
    4 - Testing others without their consent
    5 - Reduced chance of disclosure / enrolment in care
    6 - Production of fake or poor quality test kits
    7 - Other, specify:____________________________

15. **How much do you agree or disagree that:**

    "A pharmacy like this one is the best place to buy a HIV self-test kit"?

1	2	3	4	5
Strongly disagree	*Disagree*	*Undecided*	*Agree*	*Strongly agree*

    Briefly explain: ____

16. **Do you have any question or any additional information?**

    ____

17. RA's comments about the pre-test interview and pre-test counselling

18. Sale of test kit
**Did the client buy a test kit?** Yes No<br><br>**If yes, mode of delivery:**<br>1 - Over the counter<br>2 - Home delivery<br>3 - Courier (bus, etc)<br><br>**If no, comment:** ____________________

**19.**	**Queries & feedback after the participant leaves the pharmacy**		
	**Date** *(dd/mmm/yyyy)*	**Time**	**Notes**
**(a)**			
**(b)**			
**(c)**			
**(d)**			
**(e)**			
**(f)**			

Form completed by (initials): _________
