# Supplementary material for: Uptake and Acceptability of Oral HIV Self-Testing among Community Pharmacy Clients in Kenya: A Feasibility Study
Source: PLoS One. 2017 Jan 26;12(1):e0170868. doi: 10.1371/journal.pone.0170868 (PMC5268447; doi:10.1371/journal.pone.0170868)
Supplement: S2 Text — (PDF) [file pone.0170868.s004.pdf]

NJIA YA MATUMIZI

Ili upate tokeo sahihi lazima uyafuate maagizo haya kwa makini. Dakika 15 kabla ya upimaji, usile wala kunywa chochote vivyo hivyo usitumie dawa ya meno dakika 30 kabla ya upimaji.

ONYO: Iwapo una Virusi Vya Ukimwi, VVU na unatumia dawa (ART) huenda ukapata tokeo lisilo la kweli, linalo onyesha ati huna virusi.

DIRECTIONS FOR USE

You must follow the test directions carefully to get an accurate result. Do not eat or drink for at least 15 minutes before you start the test or use mouth cleaning products 30 minutes before you start the test.

WARNING: If you are HIV-positive and on HIV treatment (ARVs) you may get a false negative result.

JINSI YA KUTUMIA ORAQUICK, KIPIMA VIRUSI VYA UKIMWI, VVU, CHA KIBINAFSI / HOW TO USE THE OraQuick® HIV SELF-TEST KIT

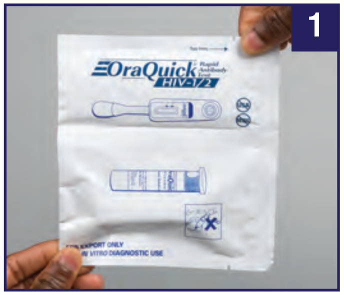

Kipima Virusi chako kina pakiti mbili zilizo unganishwa.
Your test kit contains two pouches.

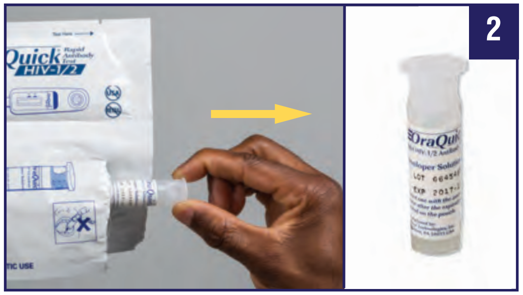

Rarua pakiti upande wenye mchoro wa Kichupa, kisha toa **kichupa.**
Tear open the pouch containing the **tube**.

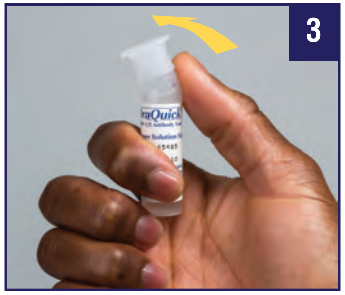

Toa kizibo/kifuniko cha kichupa.
Remove the cap.

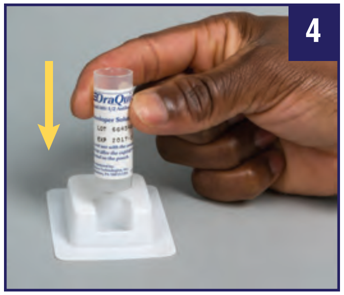

Kalisha kichupa ndani ya **Kikalio**.
Slide the tube into the **stand**.

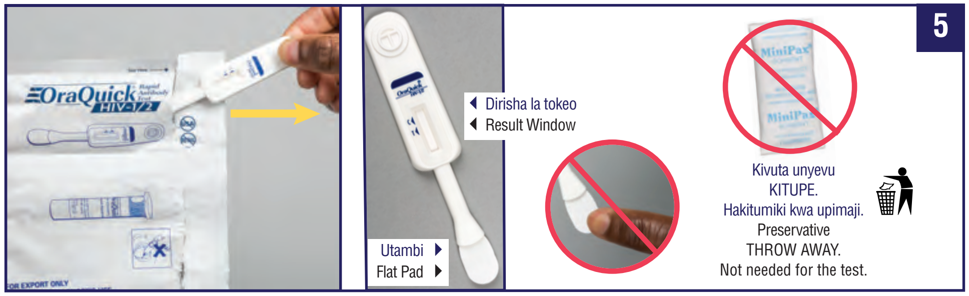

Rarua pakiti upande wenye mchoro wa **Kifaa**, kisha toa kifaa. **USIGUSE** utambi kwa vidole vyako.
Tear open pouch containing the **test device** and remove. **DO NOT** touch the flat pad with your fingers.

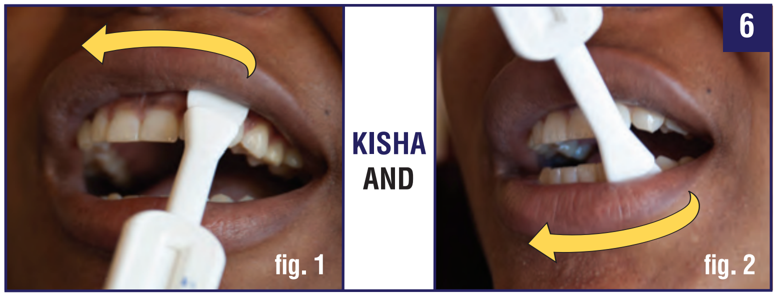

Finyilia **utambi wa Kifaa** juu ya ufizi, pangusa **ufizi wote wa juu mara moja** (fig.1) na pia **ufizi wote wa chini mara moja** (fig.2).
Press the **Flat Pad** firmly against your gum and swab it along your **upper gum once** (fig. 1) and **your lower gum once** (fig. 2).

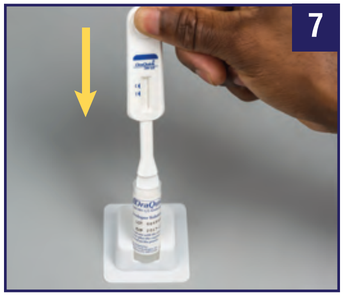

Ingiza Kifaa ndani ya kichupa hadi **Utambi** utumbukie ndani ya maji.
Put the **flat pad** all the way into the tube until it touches the bottom.

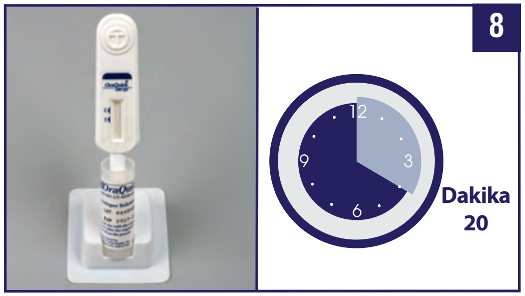

WACHA KIFAA NDANI YA KICHUPA kwa **DAKIKA 20** kabla ya kusoma Tokeo. **USISOME tokeo baada ya dakika 40.**
LEAVE IT THERE for **20 MINUTES** before reading the results. **DO NOT read the result after 40 minutes.**

KWA MATUMIZI YA UCHUNGUZI PEKEE
SIO KWA HUDUMA YA MGONJWA

FOR INVESTIGATIONAL USE ONLY
NOT FOR PATIENT CARE

Item# 3001-2864-70
rev. 08/15B

OraQuick® HIV SELF-TEST

KUTAFSIRI MATOKEO / INTERPRETING RESULTS

TOKEO CHANYA / HIV POSITIVE RESULT

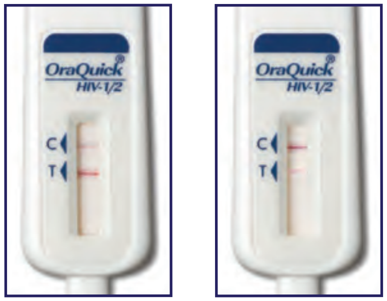

MISTARI MIWILI, hata msitari ukiwa mwembamba, inaashiria tokeo lako ni Chanya, kuna uwezekano una Virusi Vya Ukimwi, VVU na itakubidi upate upimaji zaidi.

TWO LINES, even if the line is faint, means you may be HIV POSITIVE and you need to seek additional testing.

KWA HARAKA IWEZEKANAVYO. . . / As soon as possible . . .

Tembelea kituo cha VCT au kituo cha Afya kilicho karibu nawe

Visit your nearest HIV Testing Centre or Health Facility

TOKEO HASI / HIV NEGATIVE RESULT

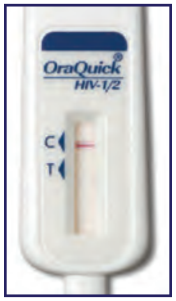

MSITARI MMOJA kwenye eneo "C" na HAKUNA msitari kwenye eneo "T", tokeo lako ni Hasi, Huna Virusi Vya Ukimwi, VVU.

ONE LINE next to the "C" and NO line next to the "T", your result is HIV NEGATIVE.

Zingatia upimaji wa mara kwa mara. Ukihisi uliambukizwa Virusi Vya Ukimwi,VVU hivi karibuni, fanya upimaji tena baada ya miezi mitatu.

Seek regular testing. If you may have been exposed to HIV, test again in 3 months.

TOKEO LISILO KUBALIKA / INVALID RESULT

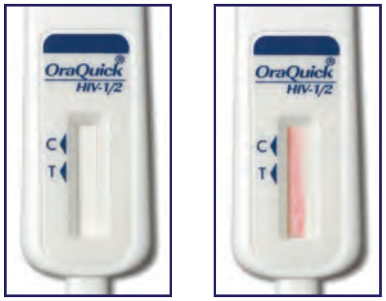

Hakuna msitari kwenye eneo "C" (hata kukiwa na msitari kwenye eneo "T"), ama kuweko kwa rangi nyekundu inayozuia kusoma tokeo, kipimo hicho si sahihi, kinapaswa kirudiwe.

Itakubidi ujipatie pakiti mpya.

No line next to the "C" (even when there is a line next to the "T"), or a red background makes it impossible to read the test, the test is not working and should be repeated.

You will need to obtain another test.

Kipimo hakikufanya kazi vyema.

Tembelea kituo cha VCT au kituo cha Afya kilicho karibu nawe upate upimaji zaidi.

The test did not work properly.

Visit your nearest HIV Testing Centre or Health Facility to test again.

KUTUPA / DISPOSE

Toa kifaa kichupani, funika kichupa, kisha tupa kila kitu ndani ya kikapu cha takataka.
Remove the test stick, put the cap on the test tube and throw away all contents in the normal trash.

Imetengenezwa Thailand kwa niaba ya:
Manufactured in Thailand for:

OraSure Technologies, Inc.
220 East First Street
Bethlehem, PA 18015
01+610-882-1820
www.OraSure.com

Item# 3001-2864 rev. 08/15B

© 2015 OraSure Technologies, Inc. • OraQuick®, logo design, and configuration are trademarks of OraSure Technologies, Inc.
