# Supplementary material for: Uptake and Acceptability of Oral HIV Self-Testing among Community Pharmacy Clients in Kenya: A Feasibility Study
Source: PLoS One. 2017 Jan 26;12(1):e0170868. doi: 10.1371/journal.pone.0170868 (PMC5268447; doi:10.1371/journal.pone.0170868)
Supplement: S3 Text — (PDF) [file pone.0170868.s005.pdf]

**PHARMACY SELF-TESTING STUDY**

Pharmacy ID: ________ Client ID: __________

Date *(dd/mmm/yyyy)*: ____ / _____ / ______

## POST-TEST PHONE QUESTIONNAIRE [DCF4] *- only for participants who buy a HST kit*

**1. How the interview was done:**

0 – Not done 1— Client called the RA 2— RA called the client 3- Face-to-face

If not done, explain: ___________________________________________________________

*Good morning/ afternoon/ Evening. I will ask your experience of conducting the self-test. I request that you answer all the questions truthfully. If you do not want to answer any of the questions you may say so and I will move on to the next question. It will take a few minutes. May I proceed?*

- ⇒ *If the participant declines, even after starting, thank them and let them know that you will not contact them again, but they can contact you later.*
- ⇒ *If the participant reports that they have not yet done the test, enquire what problem(s) they are having, encourage them to do the test as soon as possible. Find out when they think they will do the test, and agree when you can call again to complete the post-test interview.*
- ⇒ *If participant conclusively says they have decided not to use the self-test, document under Q2 below.*

**2. (a) Did the participant test?**

1 - Yes, participant tested at home
2 - Yes, participant tested at the pharmacy
3 - No, participant decided not to use the test *(Go to Q6)*

**How many days after he/she bought the kit?** _____ [0=same day; 1=following day etc]

**(b) Was test done in the presence of the counsellor?** Yes No

**(c) Did client test with partner** Yes No

**If yes, indicate client ID:** _________

**3. How easy or difficult was it to conduct the test?**

| *1* | *2* | *3* | *4* | *5* |
|---|---|---|---|---|
| *Very difficult* | *Difficult* | *Undecided* | *Easy* | *Very easy* |

Briefly describe: ___________________________________________________________

**4. (a) Did you disclose the test results to anyone?** Yes No *(Go to Q5)*

**(b) To whom did you disclose?**

1—Friend 2—Sex partner 3—Family member 4—Other, specify:_______________

*[Although participants are not required to disclose test results at any time during the study, some participants may voluntarily disclose the results during the phone interview or during other contacts. If that happens, record the results under "Results & linkage to care" section]*

**5. (a) Have you done [plan to do] a confirmatory test?**

1 - Yes
2 - No *(Go to Q6)*

**(b) What were [are] the reasons for seeking a confirmatory test?** *(select all that are mentioned)*

1 - To get a confirmation [as recommended]
2 - I did not believe the results of the self-test
3 - I don't think I did the test properly/ I messed up
4 - Other, specify:______________________________

**(c) Where did you do [plan to do] a confirmatory test?**

1 - Government hospital/ clinic
2 - Private hospital/ clinic (including NGO, CBO and faith-based facilities)
3 - KEMRI VCT/ Clinic
4 - Other VCT/ HTC centre
5 - Other, specify:______________________________

**(d)** If already done: **When did you do the confirmatory test**: _____ / _______/ _______

**(e)** If planned: **When do you plan to do the confirmatory test?**

1—This week 2—Next week 3—Later, specify: ______________________

**6. (a) How much do you agree or disagree with the statement:**

**"HIV self-test kits should be made available to the general public"?**

| *1* | *2* | *3* | *4* | *5* |
|---|---|---|---|---|
| *Strongly disagree* | *Disagree* | *Undecided* | *Agree* | *Strongly agree* |

Briefly explain: ____________________________________________________________

**7. (a) Who specifically do you think need access to HST kits?** _____________________________

**(b) Briefly explain:** ____________________________________________________________

**8. How much do you agree or disagree that:**

**"A pharmacy is the best place to buy a HIV self-test kit"?**

| *1* | *2* | *3* | *4* | *5* |
|---|---|---|---|---|
| *Strongly disagree* | *Disagree* | *Undecided* | *Agree* | *Strongly agree* |

Briefly explain: ____________________________________________________________

**9. (a) Would you like to conduct a HIV self-test on yourself again in future?**

Yes No *(Go to Q9(c)*

**(b) What did you like most about self-testing?** *( select one)*

1 - Privacy/ anonymity/ confidentiality
2 - Personal empowerment / taking charge of my own health
3 - Saves cost / no fare to the vct or clinic
4 - No pricking/ painless
5 - Saves time / no waiting in queues
6 - Other, specify:______________________________

**(c)** If NO: **What reason makes you not want to do it again?** *(select one)*

1 - Difficult to do/ complicated procedure/ unclear instructions (test kit not good)
2 - I made mistakes when doing the test (I don't think I am able)
3 - Distress when doing the test alone (need the support of a counsellor)
4 - I don't believe the results/ still have to go for confirmatory testing
5 - Other, specify:____________________________________

**10. How likely are you to recommend this test to a friend, partner or family member?**

| 1 | 2 | 3 | 4 | 5 |
|---|---|---|---|---|
| *Very unlikely* | *Unlikely* | *Undecided* | *Likely* | *Very likely* |

Briefly explain: ______________________________________________________________________

**11. What changes would you recommend in order to make self-testing better?**

______________________________________________________________________________

**12. Do you have a question or any additional information?**

______________________________________________________________________________

**TEST RESULTS & LINKAGE TO CARE**

**13. (a) Results of the HIV self-test:**

0 - Not disclosed
1 - Neg
2 - Pos
3 - Invalid

**(b) If Pos, has the client enrolled in care?**

1 - Yes Date: _____ / _______/ _______
2 - No

**14. RA's comments about the post-test interview and post-test counselling**

Form completed by (initials): _________
